# Bacterial species associated with interdigital phlegmon outbreaks in Finnish dairy herds

**DOI:** 10.1186/s12917-019-1788-x

**Published:** 2019-01-29

**Authors:** Miia Kontturi, Reijo Junni, Heli Simojoki, Erja Malinen, Eija Seuna, Kirstine Klitgaard, Minna Kujala-Wirth, Timo Soveri, Sinikka Pelkonen

**Affiliations:** 1 Department of Production Animal Medicine, Faculty of Veterinary Medicine, University of Helsinki, Paroninkuja 20, 04920 Saarentaus, Finland; 2 Veterinary Bacteriology and Pathology unit, Finnish Food Authority, Mustialankatu 3, 00790 Helsinki, Finland; 3 National Veterinary Institute, Technical University of Denmark, Kemitorvet, Building 202, 2800 Kgs. Lyngby, Denmark

**Keywords:** Interdigital phlegmon, Infectious hoof diseases, *Fusobacterium necrophorum*, *Dichelobacter nodosus*, Foot rot, Interdigital necrobacillosis, Foul-in-the-foot

## Abstract

**Background:**

Severe outbreaks of bovine interdigital phlegmon (IP) have occurred recently in several free stall dairy herds in Finland. We studied the aetiology of IP in such herds, and the association of bacterial species with the various stages of IP and herds of various morbidity of IP. Nineteen free stall dairy herds with IP outbreaks and three control herds were visited and bacteriological samples collected from cows suffering from IP (*n* = 106), other hoof diseases (*n* = 58), and control cows (*n* = 64). The herds were divided into high morbidity (morbidity ≥50%) and moderate morbidity groups (9–33%) based on morbidity during the first two months of the outbreak.

**Results:**

*F. necrophorum* subspecies *necrophorum* was clearly associated with IP in general, and *T. pyogenes* was associated with the healing stage of IP. Six other major hoof pathogens were detected; *Dichelobacter nodosus, Porphyromonas levii, Prevotella melaninogenica, Treponema* spp. and *Trueperella pyogenes.* Most of the samples of acute IP (66.7%) harboured both *F. necrophorum* and *D. nodosus.* We found differences between moderate morbidity and high morbidity herds. *D. nodosus* was more common in IP lesion in high than in moderate morbidity herds.

**Conclusions:**

Our result confirms that *F. necrophorum* subspecies *necrophorum* is the main pathogen in IP, but also *T. pyogenes* is associated with the healing stage of IP. Our results suggest that *D. nodosus* may play a role in the severity of the outbreak of IP, but further research is needed to establish other bacteriological factors behind these severe outbreaks.

## Background

During recent years, severe outbreaks of interdigital phlegmon (IP) have occurred in dairy herds in Finland, with sudden onset and of divergent morbidity. No preceding clear wound has been detected in the interdigital cleft of the IP cows. These new types of outbreaks have caused serious economic losses to affected dairy farms [1].

IP occurs usually as a sporadic infection of cattle. The herd incidence per lactation is generally 2–5% [2, 3], but studies of earlier outbreaks of IP report incidences of 17–25% during outbreaks [4, 5]. Common signs of IP are lameness; occasionally with an acute onset, a swelling of the interdigital area and the bulbs of the heels, and a fetid odour. A fissure with swollen protruding edges may appear along the interdigital cleft. In severe cases, systemic signs occur, including fever, recumbency, anorexia or decrease in milk production [6, 7]. IP reduces milk yield [8] and can necessitate early culling of the affected cow [8, 9].

Traditionally, *Fusobacterium necrophorum* is considered the major infective agent of IP [10–12] and is detected frequently in IP lesions. *F. necrophorum* is a common animal pathogen, producing several toxins that can injure tissues; leukotoxin, coded for the *lktA* gene, is considered a major virulence factor in cattle [13]. *lktA* is unique to *F. necrophorum* [14], and its detection has been used in cattle research as a reliable tool for the detection of *F. necrophorum* [15]. *F. necrophorum* is classified into two subspecies, *necrophorum* and *funduliforme.* Subspecies *necrophorum* is more frequently encountered in animal infections and in pure culture, whereas *funduliforme* is found in mixed infections and is considered less pathogenic [16].

In addition to *F. necrophorum,* several other bacteria such as, *Bacteroides melaninogenicus* [11, 12], *Dichelobacter nodosus* [7], *Porphyromonas levii* [17, 18], *Spirochetes* [5, 7], and *Trueperella pyogenes* [11] have been suggested to play a role in the pathogenesis of IP. Nevertheless, most of that research was done long ago and, for example, the taxonomical changes since then make interpretation of the results challenging. Also, a recent review describes that the role of various bacterial species in the pathogenesis of IP is still unresolved [6].

Recently the main research focus worldwide has been on digital dermatitis (DD) and treponemes and only a few studies have addressed IP and its bacteriology. However, because of numerous new type outbreaks of IP in dairy herds in Finland, we investigated the bacteriology of IP, including those bacteria earlier suggested to be involved in IP. The aim of the study was to investigate the bacteriology of IP in this new type of outbreaks; at various stages of IP, both acute and during the healing process, and compare the findings with healthy control cows. Moreover, we investigated whether these bacteriological findings differed between herds of various morbidities.

## Methods

### Herds

During 2012–2015 we carried out a research project on infectious hoof diseases in Finland. As a part of the project, we made several farm visits to privately owned dairy herds affected by outbreaks of IP. Of the farms visited, 19 fulfilled the criteria for an outbreak of IP; at least three observed cases of IP within 1 week, and no previous history of IP in the herd for 10 years. The outbreak herds were later divided into two categories based on the incidence of IP within 2 months of the outbreak. Furthermore, we collected samples from control cows of three non-outbreak herds (IP free herds). All herds studied were housed in free stalls. The average herd size was 75 lactating cows (range 31–140, median 62) and the average milk yield was 9234 kg (8000–10,914 kg, median 9219 kg).

### Cows

The primary selection criteria for inclusion of a cow in the study were lameness, prolonged lying-time, or a ‘trouble report’ from an automatic milking system. In the outbreak herds, we collected samples mainly from cows that had IP, but also from lesions apparently infected with bacteria. Such lesions included DD, interdigital dermatitis (ID), white line abscesses and sole ulcers. The IP lesions were classified as acute IP or healing IP. The diagnosis of acute IP was made if a symmetric swelling and possible ulceration appeared in the interdigital cleft. Healing IP was identified as proliferation tissue or apparent scar formation in the affected region. DD diagnosis was made according to Döpfer et al. [19]. We also sampled 1–5 control cows per IP outbreak herd. These were non-lame cows with no sign of IP, DD, ID, sole ulcer, or white line disease, and are hereafter referred to as control cows (IP herd). In control herds, we sampled 4–8 cows in each herd using the same criteria as for control cows (IP herd).

We sampled a single hoof from all control cows, but from 11 cows with IP or DD we took samples from two separate feet. Five outbreak herds were visited 2 or 3 times. During these visits, 10 IP cows were sampled repeatedly 2 or 3 times 11–34 days after the first sampling. These samples were additional and not included in the total number of hoof samples (total *n* = 228). These resampled IP cows had clinical signs of IP at all sampling times. Table 1 presents the number of study herds of various morbidities, numbers of sampled cows and hoof samples in various disease groups.

**Table 1:** Dairy herds, cows and bacteriological samples of a study of interdigital phlegmon outbreaks in Finland

	Herd (n)	Cow (n)	Control (IP free herd)	Control (IP herd)	Acute IP	Healing stage IP	Other hoof disease
Number of herds	19	217					
High morbidity herd	7	65		13 (28.9%)	27 (45%)	11 (27.5%)	14 (26.4%)
Moderate morbidity herd	12	133		32 (71.1%)	33 (55%)	29 (72.50%)	39 (73.6%)
Non-outbreak herd	3	19	19 (100%)				
Number of cows		217	19/217 (8.8%)	45/217 (20.7%)	60/217 (27.7%)	40/217 (18.4%)	53/217 (24.4%)
Cows with antibiotic treatment							
None		151 (69.6%)	19 (100%)	45 (100%)	31 (51.7%)	7 (17.5%)	49 (92.5%)
Current		37 (17.1%)	0 (0%)	0 (0%)	21 (35%)	15 (37.5%)	1 (1.9%)
Previous		29 (13.4%)	0 (0%)	0 (0%)	8 (13.3%)	18 (45%)	3 (5.7%)
		Hoof sample (n)	Control (IP free herd)	Control (IP herd)	Acute IP	Healing stage IP	Other hoof disease
Number of hoof samples		228^a^	19 (8.3%)	45 (19.7%)	65^a^ (28.5%)	41^a^ (18.0%)	58^a^ (25.4%)
Front feet		25 (11.0%)	0 (0%)	5 (11.1%)	12 (18.5%)	5 (12.2%)	3 (5.2%)
Hind feet		203 (89.0%)	19 (100%)	40 (88.9%)	53 (81.5%)	36 (87.8%)	55 (94.8%)
Hoof sample with antimicrobial treatment							
None		159 (69.7%)	19 (100%)	45 (100%)	36 (55.4%)	7 (17.1%)	52 (89.7%)
Current		38 (16.7%)	0 (0%)	0 (0%)	21 (32.3%)	16 (39.0%)	1 (1.7%)
Previous		31 (13.6%)	0 (0%)	0 (0%)	8 (12.3%)	18 (43.9%)	5 (8.6%)
Number of PCR tests		Hoof sample (n)	Control (IP free herd)	Control (IP herd)	Acute IP	Healing stage IP	Other hoof disease
*Fusobacterium necrophorum*		205	19 (100%)	43 (95.6%)	52 (80.0%)	37 (90.2%)	54 (93.1%)
*Dichelobacter nodosus*		205	19 (100%)	43 (95.6%)	52 (80.0%)	37 (90.2%)	54 (93.1%)
*Porphyromonas levii*		142	19 (100%)	41 (91.1%)	49 (75.4%)	33 (80.5%)	Not analyzed
*Prevotella melaninogenica*		142	19 (100%)	41 (91.1%)	49 (75.4%)	33 (80.5%)	Not analyzed
*Treponema* group 2 & 3		168	19 (100%)	42 (93.3%)	39 (60.0%)	36 (87.8%)	32 (59.3%)
*Trueperella pyogenes*		205	19 (100%)	43 (95.6%)	52 (80.0%)	37 (90.2%)	54 (93.1%)

^a^Two feet were sampled from 11 cows (5 acute IP, 1 healing stage IP, 5 other hoof diseases)

Numbers of sampled cows, numbers of hoof samples and a possible antimicrobial treatment in various disease groups; control cows in a herd with no outbreak of interdigital phlegmon, IP (IP free herd), control cows in a herd with an outbreak of IP (IP herd), acute interdigital phlegmon (Acute IP), IP at healing stage (Healing IP) and hoof diseases other than IP (Other). The group “Other” included hoof samples from digital dermatitis, interdigital dermatitis, white line abscess and sole ulcer. With antibiotic treatment, “None” signifies no current or previous antibiotic treatment during last month, “Current” signifies current antibiotic treatment or treatment within 6 days before sampling and “Previous” means previous treatment with antibiotics within 7–30 days prior to the sampling. “Number of PCR tests**”** column means the number of samples that were successfully amplified in PCR

Of the sampled cows (*n* = 217) selected for the study, 58.5% were Ayrshire and 41.5% Holstein. Moreover 4.6% were heifers, 41.5% first parity cows, 22.1% second parity, 29.5% third or more parity cows and 36.9% were on early lactation (1–120 days in milk, DIM), 53.0% late lactation (121–305 DIM) and 7.8% were either dry cows or heifers. Information on parity and lactation stage was absent for 5 cows (2.3%).

### Sampling methods and materials

Two veterinarians (MK, RJ) experienced in hoof diseases of cattle, evaluated the general condition and hoof health of the cows prior to sampling and recorded clinical diagnosis and antimicrobial treatment history. Every hoof was photographed at sampling, and diagnoses were standardized between the two veterinarians by evaluating some of the photographs together.

The sampling took place in a trimming chute; we lifted the foot up and spread the claws with an extensor. Then we washed the distal foot carefully with a hose, spouted with saline solution, and dried it with gauze. We collected the bacterial samples from the inflamed region using sterile swabs (FLOQSwabs), used them immediately for culturing, and took cytobrush samples from the same region for PCR analysis. We placed the cytobrushes (Medscand Medical Cytobrush Plus, CooperSurgical Inc., Germany) in sampling tubes (Micro tube 2 mL, Sarstedt, Germany) and froze them to – 20 °C in 24 h. We sampled the control cows similarly from the interdigital cleft. All bacterial samples in this study are hereafter referred as hoof samples. If needed, the farm veterinarian treated IP and DD cows with severe signs after sampling.

### Bacteriological culture

During the farm visits, we set up a field laboratory at the farm. It included culture media, disposable plastic loops (10 μL, Mekalasi Oy, Helsinki, Finland) and equipment to maintain anaerobic conditions. Fastidious Anaerobe Agar, FAA (LabM, Lancashire, UK) and Fusobacterium Neomycin Vancomycin, NV agar [20] were used for primary culture. NV media were provided by Kokkola laboratory ((Maintpartner OY, Kokkola, Finland) or the Finnish Food Safety Authority (Evira, Helsinki, Finland). Agar plates were prereduced in Genbox containers (Biomerieux, France). The agar plates were sealed to maintain anaerobic conditions (BD GasPak EZ, Becton, Dickinson and Company, USA and GENpag anaer, Biomerieux, France) within 2 hours of sampling and incubated anaerobically for 2 days at 37 °C.

### Isolation and identification of *Fusobacteriumnecrophorum*

From cultures we picked greyish, umbonate colonies of various shapes and sizes typical of spp. *necrophorum*, and smaller, yellowish, and waxy colonies typical of spp. *funduliforme*. Both colony types expressed strong beta-haemolysis on FAA and NV agars. The colonies were identified using conventional bacteriological methods to species and subspecies level [20], and verified using PCR assays for *lkt*A and *haemagglutinin* (Table 2). Isolates were stored below − 70 °C for further characterisation.

**Table 2:** PCR oligos and reaction conditions of a study of interdigital phlegmon outbreaks in Finnish dairy herds

PCR assay	Oligos	Annealing temperature (°C)	PCR product (bp)	Reference
*Dichelobacter nodosus*	16S(F2): CGGGGTTATGTAGCTTGC	60	783	[43]
	16S(R2): TCGGTACCGAGTATTTCTACCCAACACCT			
*F. necrophorum leucotoxin*	LT3 F: GGAGTAAGAGCAACTATGGGAGCAGCTAC	60	360	[44]
	LT3 R: CCCAATCCACCTTTTACAGCAGCTCG			
*F. necrophorum hemagglutinin*	HAEM F: CATTGGGTTGGATAACGACTCCTAC	55	286	[45]
	HAEM R: CAATTCTTTGTCTAAGATGGAAGCGG			
*Trueperella pyogenes* pyolysin	PLO F: TCATCAACAATCCCACGAAGAG	60	150	[46]
	PLO R: TTGCCTCCAGTTGACGCTTT			
Universal 16S	27f YM: 5’-AGAGTTTGATYMTGGCTCAG-3′	53	~ 1500	[47]
	1492 r: 5’-TACCTTGTTACGACTT-3′			
*Porphyromonas levii*				
PORP01F01	GACCAAATCGTCGTACTTGACAAA	66.2	75	
PORP01R01	GCCTCGGCTGGCAGTAAG	66.4		
PORP01P01FAM	ACTCTCATGGTTGCCTACTTCTACAATCTTTCC	71.3		
*Prevotella melaninogenica*				
PREV01F01	CCCGGCTGTTTAGAATACTTTGTCA	67.8	152	
PREV01R02	CTTTGCATGGGTGGTGTTGAT	67.2		
PREV01P01FAM	AATTAATCGTCGTCCGATATCACCACATACAGAG	73.4		
*Treponemas*				
Group 1 (*T. medium*/*T. vincentii* –like)	TmF 5′-GAATGCTCATCTGATGACGGTAATCGACG-3′	68	472–500	[21]
	TmR 5′-CCGGCCTTATCTAAGACCTTCTACTAG-3′			
Group 2 (*T. phagedenis*-like)	TbF 5′-GAAATACTCAAGCTTAACTTGAGAATTGC-3′	64	400	[21]
	TbR 5′-CTACGCTACCATATCTCTATAATATTGC-3′			
Group 3 (*T. denticola* /*T. putidum*-like)	TpF 5′-GGAGATGAGGGAATGCGTCTTCGATG-3′	67	475	[21]
	TpR 5′-CAAGAGTCGTATTGCTACGCTGATATATC-3′			
Universal 16S	16S F 5′-AGAGTTTGATCCTGG-3′	57	1526	[48]
	16S R 5′-TACCTTGTTACGACTT-3′			

### DNA extraction from the cytobrush samples

Total DNA was extracted from cytobrush samples with Qiagen Blood and Tissue Column kit (Qiagen Gmbh, Germany) following the manufacturer’s instructions. The samples were eluted in 100 μL EB and stored at − 20 °C. An aliquot of 2 μL was used as a template for PCR amplification. Bovine DNA in the preparations did not block the detection of bacterial target DNA by PCR. The PCR assays are listed in Table 2.

### PCR for *Fusobacterium necrophorum*, *Dichelobacter nodosus* and *Trueperellapyogenes*

The PCR analyses were performed at the Finnish Food Safety Authority Evira. Of the 228 samples analyzed, 205 samples were successfully amplified. The PCR assays, oligos and conditions for PCR are shown in Table 2. PCR reactions consisted of 0.5 μM of each oligo, 200 μM dNTP (Thermo Fisher Scientific), 1.0 U Dynazyme polymerase, 1.5 mM MgCl_2_ and 2 μL template in Dynazyme F-511 buffer (Thermo Fisher Scientific). PTC Thermal cycler (Thermo Fisher Scientific™) was used for ampilification. For *lktA* gene, the thermal profile consisted of 95 °C for 2 min followed by 35 cycles of 95 °C for 30 s, 60 °C for 30 s, 72 °C for 40 s, with a final extension at 72 °C for 5 min. For *haemagglutinin* gene, the thermal profile was 95 °C for 2 min, followed by 35 cycles of 95 °C for 30 s, 55 °C for 15 s and 72 °C for 30 s, with a final extension at 72 °C for 5 min. The PCR products were separated and visualized using electrophoresis and SybrSafe in 1.5% agarose gel.

### PCR for *Porphyromonaslevii* and *Prevotella melaninogenica*

The PCR analyses were performed at ThermoFisher Scientific Vantaa, Finland. Control (IP herd and IP free herd) and IP samples (*n* = 142) were analysed. All PCR reactions contained 0.5 μM of primers and 0.25 μM of probes in 20 μL of final PCR volume. QuantStudio 5 Real-Time PCR System (Thermo Fisher Scientific™) was used for thermal cycling. The thermal profile consisted of 95 °C for 10 min followed by 40 cycles of 95 °C for 5 s, and 60 °C for 1 min. In-house programs were applied to design qPCR oligo sequences for *P. levii* and *P. melaninogenica* used in this study (Table 2). Inclusivity and exclusivity were confirmed in silico using all RefSeq (NCBI Reference Sequence Database) bacterial genomes as reference sequence data.

Commercial genomic DNA (gDNA) stocks from *P. melaninogenica* DSM26980 and *P. levii* DSM23370 were measured using a Qubit Fluorometer (Qubit® 2.0 Fluorometer, Thermo Fisher Scientific™) and gDNA copy numbers were calculated using DNA Copy Number and Dilution Calculator (Thermo Fisher Scientific™). Both oligo sets were multiplexed with an internal amplification control (IAC) oligos and template DNA (eliminates false-negative results due to inhibition of the reaction) and compared to the singleplex reactions using a genomic DNA dilution series in triplicate. Amplification efficiency for both oligo sets was calculated from the multiplex reactions. No-template controls (NTC) were run with each multiplex to screen potential oligo cross-reactions. Sensitivity of the oligo sets was tested using a doubling dilution series of genomic DNA in 8 replicates. Specificity of both oligo sets was tested using the non-target panel of several bacteria. DNA samples were analysed with the two oligo sets using 2 μL of DNA. Positive controls and NTC’s were included into each run.

### PCR for *Treponema*

The PCR analyses were performed at Denmark Technical University. Altogether 168 cytobrush samples had enough DNA for the analysis. An initial PCR step using a universal bacterial oligo pair encompassing most of the 16S rRNA gene [21] was followed by nested PCR analysis using oligos specific for the three DD *Treponema* phylogroups as described by Evans et al. [21] (Table 2). In all PCR assays, a 25 μL reaction mixture contained 1.25 U AmpliTaq DNA polymerase (Applied Biosystems, CA, USA), 1.5 mM (universal oligos) or 3 mM (group specific oligos) MgCl_2_ Solution (Applied Biosystems, USA), 100 μM of each dNTP (Amersham Biosciences, NJ, USA), 0.2 μM of each specific oligo, and 1 μL of the template in PCR Buffer II (Applied Biosystems, USA). Thermal cycling was performed in a T3 thermocycler (Biometra, Göttingen, Germany) as described by Evans et al. [21]. In each assay, water served as a negative control, and genomic DNA from each of the three *Treponema* groups as positive control. PCR products were separated on a 2% E-gel (Invitrogen, Carlsbad, 92,008 CA, USA), and visualized by UV fluorescence.

### Bacterial controls

The following type strains were used as controls in the PCR assays: *D. nodosus* ATCC 25549, *F. necrophorum* ssp. *necrophorum* ATCC 25286, *F. varium* ATCC 8501, *F. necrophorum* ssp. *funduliforme* DSM 19678, *T. pyogenes* ATCC 19411D, *P. levii* (DSM23370) and *P. melaninogenica* (DSM26980), *T. vincentii* (ATCC 35580), *T. phagedenis* (ATCC 27087) and *T. denticola* (ATCC 3320). Our own *Arcanobacterium haemolyticum* isolate served as a negative control for *T. pyogenes* pyolysin.

### Statistical analysis

The bacteriological results and data recorded during the herd visits were entered Excel spreadsheets and the statistical analyses were carried out using Stata IC version 15.0 (Stata Corporation, Texas, USA). A *p*-value of < 0.05 was considered statistically significant. The repeated samples were excluded from statistical analyses.

Two groups of cows served as controls in our study; control in IP free herd (*n* = 19) and in IP herd (*n* = 45), and were tested for statistical difference using chi square. All hoof samples were divided into four disease categories; 1) control, 2) acute IP, 3) healing IP, and 4) other hoof diseases. Antimicrobial treatments were divided into three categories; 1) no current or previous antimicrobial treatment during last month, 2) current antimicrobial treatment or treatment within 6 days before sampling and 3) previous treatment with antimicrobials within 7–30 days prior to the sampling. The outbreak herds (*n* = 19) were divided into two categories 1) herds of high morbidity; ≥50% of the cows having IP and 2) herds of moderate morbidity; 9–33% of the cows with IP during the first 2 months of the outbreak. No herds had morbidity between these figures.

The effect of antimicrobial treatment to each bacterium was tested separately with a logistic regression model. The dependent variable was each bacterium separately and independent variables were disease categories 1–4 and antimicrobial treatment categories 1–3. Herd was included as a random factor in these models.

The possible association of culture results of fusobacteria and IP were tested using chi-squared test. The possible association of bacteria in IP samples and high or moderate morbidity outbreak of IP were tested using Fisher exact test; only cows without antimicrobial treatment were included in the analysis.

We studied the association of disease categories and various bacteria (*n* = 6) in a multinomial logistic regression model. The herd had no effect on the results and was not included to the final model. The outcome of the model was disease categories (control, acute IP, healing IP) and variables were *F. necrophorum, D. nodosus, T. pyogenes, Treponema, P. levii* and *P. melaninogenica* (all dichotomous, no presence/presence). The group of other hoof diseases was excluded from this analysis.

## Results

### Association of *Fusobacterium necrophorum *isolates in different disease categories

**Fig. 1:**
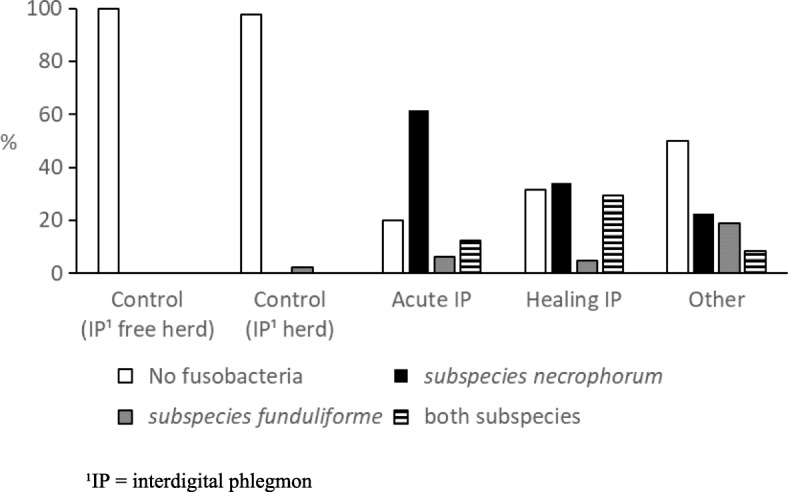
Detection of *Fusobacterium necrophorum* ssp. *necrophorum* and ssp. *funduliforme* by culture in hoof samples from various disease categories. Samples (*n* = 228) were collected from control cows (IP free herd, *n* = 19), control cows (IP herd, *n* = 45), acute interdigital phlegmon (Acute IP, *n* = 65), during the healing process of IP (Healing IP, *n* = 41) and from other hoof diseases than IP, including digital dermatitis, interdigital dermatitis, white line abscess and sole ulcer (Other, *n* = 58)

*F. necrophorum* ssp. *necrophorum* was detected by culture in 48/65 (73.8%) of the samples from acute IP and in 26/41 (63.4%) from healing IP and was clearly associated with IP (*p* < 0.01) when both IP groups (*n* = 106) were combined and compared with controls (*n* = 64). All the *F. necrophorum* isolates, including both subspecies *necrophorum* and *funduliforme*, possessed the *lktA* gene. Figure 1 shows the prevalence of cultured fusobacteria in various disease categories; control cows (IP free herd, *n* = 19), control cows (IP herd, *n* = 45), acute IP (*n* = 65), healing IP (*n* = 41), other hoof diseases (*n* = 58).

The group of other hoof diseases (*n* = 58) included samples from cases of DD, ID, sole ulcer and white line abscesses. In 20 DD samples, *F. necrophorum* ssp. *necrophorum* was detected in 7 (35.0%) samples. In other hoof diseases, including ID, white line abscesses and sole ulcers, ssp. *necrophorum* was detected in 11/38 (28.9%) of the samples.

### Isolation of *Fusobacterium necrophorum *from repeated samples

The resampled hooves were culture negative for *F. necrophorum* ssp. *necrophorum* in a first sampling, but both were positive subsequently. One sample was positive at both samplings and seven samples were negative at the second sampling. One cow was sampled three times and after being positive for *F. necrophorum* ssp. *necrophorum* at the first sampling, it was negative at the second and positive at the third sampling. All cows except one were treated with antimicrobials between sampling times.

### PCR results

We obtained PCR results for *D. nodosus, F. necrophorum* and *T. pyogenes* from 205 hoof cytobrush samples*, P. levii* and *P. melaninogenica* from 142 and *Treponema* from 168 samples. Figure 1 shows the number of successful PCR tests in each disease category. Of 168 *Treponema* samples, 93 (55.4%) were positive for universal *Treponema* primer. None of the samples was positive for *Treponema* group 1 (*T. medium/ T. vincentii*-like). However, 28/168 (16.7%) were positive for *Treponema* group 2 (*T. phagedenis*-like) and 16/168 (9.5%) for *Treponema* group 3 (*T. putidum/ T. denticola*-like). *Treponema* group 3 was always detected simultaneously with *Treponema* group 2. Of these 16 samples were 4 acute IP, 3 healing IP, 8 DD and 1 other hoof disease.

### PCR results for control cows

*D. nodosus* was detected from 9/19 (47.4%) control cows (IP free herd) and 21/43 (48.8%) control cows (IP herd), *F. necrophorum* was detected in 0/19 (0%) and 4/43 (9.3%) of samples, *P. levii* in 1/19 (5.6%) and 3/41 (7.3%), *P. melaninogenica* in 0/19 (0%) and 2/41 (4.9%), *Treponema group* 2 and 3 in 4/19 (21.1%) and 6/42 (14.3%), and *T. pyogenes* in 1/19 (5.3%) and 0/43 (0%). No statistical differences were evident between the control groups regarding the bacteria detected and therefore data for each control group were combined for statistical analyses.

### PCR results for samples of IP and other hoof diseases

**Fig. 2:**
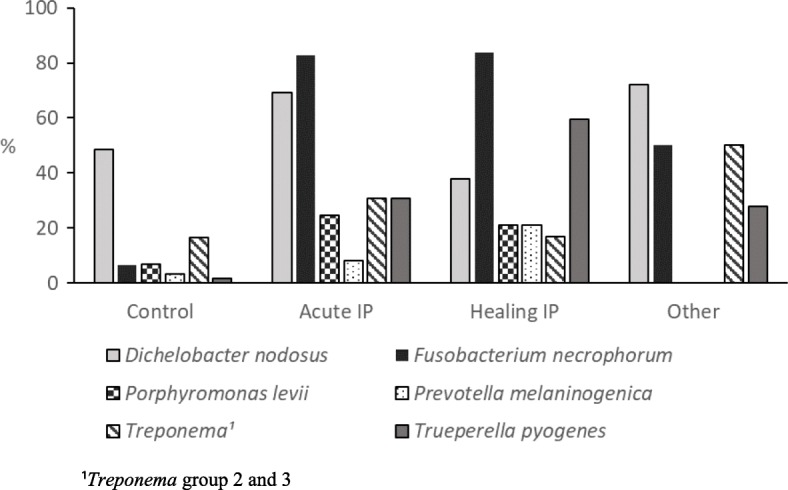
Detection of bacteria by PCR in hoof samples from various disease categories. The disease categories included; control cows (*n* = 62), acute interdigital phlegmon (Acute IP, *n* = 52), IP in a healing stage (Healing IP, *n* = 37) and other hoof diseases than IP (Other, *n* = 54). The group other hoof diseases included hoof samples from digital dermatitis, interdigital dermatitis, white line abscess and sole ulcer. Total number of hoof samples is 205, except with *P. levii* and *P. melaninogenica* (142) and *Treponema* group 2 and 3 (168)

Figure 2 presents the results of PCR analysis for various disease categories; control cows (*n* = 62), acute IP (*n* = 52), healing IP (*n* = 37), and other hoof diseases (*n* = 54). *P. levii* and *P. melaninogenica* were not analysed among the group of other hoof diseases. Several bacterial species were detected by PCR in numerous hoof samples (Table 3). The control cows were either PCR negative (26/59; 44.1%), or harboured *D. nodosus* alone (16/59; 27.1%) or in combination with *Treponema* group 2 and 3 (9/59; 15.3%). In most acute IP samples (24/36; 66.7%), *F. necrophorum* and *D. nodosus* were detected. They occurred with *P. levii* (4/36; 11.1%) or *Treponema* group 2 and 3 (4/36; 11.1%), and *F. necrophorum* alone was combined with *T. pyogenes* (4/36; 11.1%). For the healing stage of IP, the most frequently detected combinations were *F. necrophorum* and *T. pyogenes* (6/33, 18.2%), *F. necrophorum* alone (3/33; 9.1%) and *F. necrophorum*, *T. pyogenes* and *P. levii* (3/33, 9.1%).

**Table 3:** Combinations of bacterial species detected by PCR in various disease categories in interdigital phlegmon outbreaks in Finnish dairy herds

Bacterial combination	Control	Acute IP	Healing IP
n	59	36	33
No detected bacteria	26	2	
*P. melaninogenica*			1
*P. levii*	3		
*Treponema* ^a^			1
*T. pyogenes*	1	1	
*D. nodosus*	16		2
*D. nodosus, P. melaninogenica*	1		
*D. nodosus, Treponema*	9	2	
*D. nodosus, Treponema, P. levii*		1	
*D. nodosus, Treponema, T. pyogenes*			1
*F. necrophorum*		1	3
*F. necrophorum, P. melaninogenica*			1
*F. necrophorum, P. levii*	1		2
*F. necrophorum, Treponema*		1	1
*F. necrophorum, T. pyogenes*		4	6
*F. necrophorum, T. pyogenes, P. melaninogenica*			2
*F. necrophorum, T. pyogenes, P. levii*			3
*F. necrophorum, T. pyogenes, P. levii, P. melaninogenica*			1
*F. necrophorum, T. pyogenes, Treponema*			1
*F. necrophorum, D. nodosus*	1	7	2
*F. necrophorum, D. nodosus, P. melaninogenica*			1
*F. necrophorum, D. nodosus, P. levii*		4	
*F. necrophorum, D. nodosus, P. levii, P. melaninogenica*		1	
*F. necrophorum, D. nodosus, Treponema*		4	
*F. necrophorum, D. nodosus, P. melaninogenica, Treponema*	1	1	
*F. necrophorum, D. nodosus, T. pyogenes*		2	2
*F. necrophorum, D. nodosus, T. pyogenes, P. melaninogenica*			1
*F. necrophorum, D. nodosus, T. pyogenes, P. levii*		1	1
*F. necrophorum, D. nodosus, T. pyogenes, P. levii, P. melaninogenica*		1	
*F. necrophorum, D. nodosus, T. pyogenes, Treponema*		2	1
*F. necrophorum, D. nodosus, T. pyogenes, P. levii, Treponema*		1	

^a^*Treponema* includes *Treponema* group 2 and 3

Various disease categories are control cows, interdigital phlegmon (IP) in an acute stage (Acute IP), and IP during a healing process (Healing IP). Total number of control and IP hoof samples is 128

### Association of disease categories and bacterial species

We investigated the association of control samples, acute IP, and healing IP with the bacterial species detected by PCR (Table 4). *F. necrophorum* was associated distinctively with both stages of IP (*p* < 0.01). *T. pyogenes* was found more often with the healing IP (*p* = 0.01), but only a trend existed in the group of acute IP samples. Antimicrobial treatment affected detection of *D. nodosus* (current treatment OR = 0.2, p = 0.01, previous treatment OR = 0.1, p < 0.01) and *Treponema* group 2 and 3 (current treatment OR = 0.1, p < 0.01, previous treatment OR = 0.1, *p* = 0.03), but not detection of other bacteria.

**Table 4:** The multinomial logistic regression model for the association of various disease categories and presence of bacteria in outbreaks of interdigital phlegmon in Finnish dairy herds

Disease categories	*n*	RRR^a^	*p*-value	95% CI^b^
Control cows	59	Base outcome		
Acute IP	36			
*Dichelobacter nodosus*		2.1	0.36	0.44–9.88
*Fusobacterium necrophorum*		74.9	< 0.01	14.31–391.71
*Porphyromonas levii*		1.7	0.62	0.22–12.43
*Prevotella melaninogenica*		0.7	0.80	0.04–12.67
*Treponema*^c^		3.8	0.11	0.75–19.33
*Trueperella pyogenes*		10.8	0.06	0.91–127.48
Constant^d^		0.04	< 0.01	0.01–0.15
Healing IP	33			
*Dichelobacter nodosus*		0.4	0.26	0.08–1.95
*Fusobacterium necrophorum*		58.4	< 0.01	10.29–332.00
*Porphyromonas levii*		1.1	0.96	0.13–8.73
*Prevotella melaninogenica*		3.0	0.44	0.19–47.02
*Treponema*^c^		2.2	0.40	0.35–13.76
*Trueperella pyogenes*		22.4	0.01	2.01–249.04
Constant^d^		0.08	< 0.01	0.02–0.25

^a^RRR = relative risk ratio

^b^95% CI = 95% confidence interval

^c^*Treponema* group 2 and 3

^d^Constant is a baseline relative risk for each outcome

The disease categories were control cows, acute interdigital phlegmon (Acute IP) and IP in a healing stage (Healing IP). The herd had no effect on the results. In this model, the number of the hoof samples is 128

### Bacterial findings in high and moderate morbidity herds

**Fig. 3:**
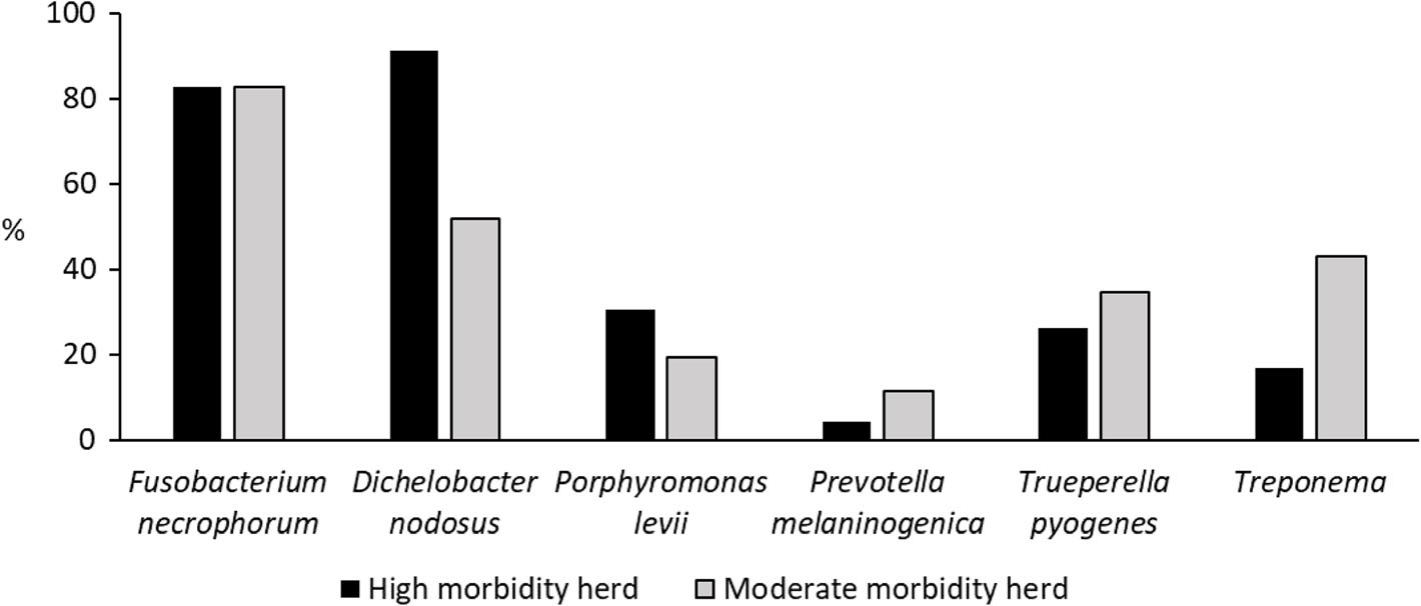
PCR results for hoof samples from acute interdigital phlegmon (IP) in herds with various morbidity. We visited high morbidity (morbidity ≥50% during the first two months of the outbreak) and moderate morbidity (morbidity 9–33%) herds. Number of hoof samples is 52, except with *P. levii* and *P. melaninogenica* (*n* = 49) and *Treponema* (*n* = 39). *Treponema* includes *Treponema* group 2 and 3

Of 19 outbreak herds, in 7 herds the morbidity was high (morbidity ≥50% during first 2 months of the outbreak) and 12 herds moderate (morbidity 9–33%). No herds had morbidity of 34–49%. We found no differences in detected bacteria in control samples of herds of various morbidity. We focused on acute IP samples and compared their bacteriology between these 7 high morbidity herds and 12 moderate morbidity herds. Bacterial species detected by PCR in hoof samples from acute IP in high and moderate morbidity herds are presented in Fig. 3 and combinations of bacterial species detected in Table 5.

**Table 5:** Combinations of bacterial species detected by PCR in hoof samples from acute interdigital phlegmon (*n* = 36) in high morbidity (≥50%) and moderate morbidity (9–33%) Finnish dairy herds

Bacterial combination	High	Moderate
n	17	19
No detected bacteria		2
*T. pyogenes*		1
*D. nodosus and Treponema* ^a^	1	1
*D. nodosus, Treponema, P. levii*	1	
*F. necrophorum*		1
*F. necrophorum, Treponema*		1
*F. necrophorum, T. pyogenes*	1	3
*F. necrophorum, D. nodosus*	7	
*F. necrophorum, D. nodosus, P. levii*	3	1
*F. necrophorum, D. nodosus, P. levii, P. melaninogenica*		1
*F. necrophorum, D. nodosus, Treponema*		4
*F. necrophorum, D. nodosus, P. melaninogenica, Treponema*		1
*F. necrophorum, D. nodosus, T. pyogenes*	2	
*F. necrophorum, D. nodosus, T. pyogenes, P. levii*		1
*F. necrophorum, D. nodosus, T. pyogenes, P. levii, P. melaninogenica*	1	
*F. necrophorum, D. nodosus, T. pyogenes, Treponema*	1	1
*F. necrophorum, D. nodosus, T. pyogenes, P. levii, Treponema*		1

^a^*Treponema* group 2 and 3

First, we analysed the association of culture results of fusobacteria in various morbidity herds. The presence of 2 *F. necrophorum* subspecies in acute IP samples from high (*n* = 31) and from moderate morbidity herds (*n* = 34) did not differ (*p* = 0.24); of these samples, no fusobacteria were detected in 9 (29.0%) samples from high and in 4 (11.8%) samples from moderate morbidity herds. Subspecies *necrophorum* was detected in 18 samples (58.1%) from high and in 22 (64.7%) samples from moderate morbidity, ssp. *funduliforme* in 2 samples from both morbidity groups (6.5 and 5.9% respectively), and both subspecies in 2 samples (6.5%) from high and in 6 (17.7%) from moderate morbidity herds. Subsequently we compared the association of other bacteria in various morbidity herds; presence of *D. nodosus, F. necrophorum*, *P. levii*, *P. melaninogenica*, *Treponema* group 2 and 3, and *T. pyogenes* detected by PCR in acute IP samples from high and moderate morbidity herds is presented in Fig. 3.

The most common combination, *F. necrophorum* and *D. nodosus*, was found in 14/17 (82.4%) samples of acute IP from high and 10/19 (52.6%) samples from moderate morbidity herds (Table 5). *D. nodosus* was more often detected in IP in high than moderate morbidity herds (*p* = 0.05, *n* = 35).

## Discussion

*F. necrophorum* was found in this study as the main pathogen in IP. This is in line with previous studies [10–12]. Based on our results, it was ssp. *necrophorum* that was clearly associated with IP. We also detected *F. necrophorum* in DD and in other hoof diseases, but less frequently than in IP. Similarly, fusobacteria are detected in DD lesions in other studies [22, 23].

*F. necrophorum* is a normal inhabitant in the rumen of cattle [24]. Occasionally, it can be detected in the faeces, and thus it contaminates the environment [25]. In a study of DD microbiome, small number of fusobacteria were detected on healthy hooves [26]. Similarly, in our study *F. necrophorum* ssp. *necrophorum* was not detected on the skin of healthy hooves, even when a severe IP outbreak was evident in the herd. This indicates that *F. necrophorum* does not colonize the intact skin in large numbers. A moist environment or possible trauma has been mentioned as predisposing factors for IP in previous studies [7, 27]. Interestingly, in most of our acute IP study cows no hoof trauma was visible. In most of the study herds the free stall was also reasonably new and well-managed. As a result, we can speculate that *F. necrophorum* may have to interact with other bacteria to invade to the subcutaneous tissue in the interdigital cleft.

Unexpectedly in repeated sampling, fusobacteria were cultivated from IP lesions even though cows had been treated with antimicrobials and IP was at the healing stage. The clinical signs appeared to diminish after beginning of antimicrobial treatment, but *F. necrophorum* remained in the affected region. However, our bacteriological methods were not quantitative and therefore, we do not know the number of detected bacteria and whether the amount had diminished or not. In a small pilot study of outbreaks of IP in two herds, susceptibility of 27 *F. necrophorum* isolates to penicillin, tetracycline, cefuroxime and cefotaxime was determined by E-test. All isolates were found susceptible to tested antimicrobials [28]. Also other study reports that antimicrobial resistance is not characteristic of *F. necrophorum* in IP [29].

We detected *D. nodosus* from healthy hooves, IP and other hoof diseases. In most of the acute IP samples (66.7%), both *F. necrophorum* and *D. nodosus* were detected*.* A significant association was established with the presence of *D. nodosus* in IP lesions and high morbidity outbreak in the herd. This could indicate that the presence of *D. nodosus* affects the severity of IP. *D. nodosus* is associated with ID [30] and DD [30–33] and detected in healthy hooves [30]. It is hypothesised that *D. nodosus* could break down the epidermal barrier, creating a suitable environment for secondary invaders [32]. A recent study also suggests *D. nodosus* as a potentially important pathogen in DD [23]. Our qualitative investigation does not take account of the numbers of bacteria, which might differ in IP lesions compared with healthy hooves.

*P. levii* and *T. pyogenes* are detected with *F. necrophorum* in various cattle infections and evidence of interactions and possible synergism between these species is reported [34–37]. IP is induced using field strains of *F. necrophorum* and *B. melaninogenicus* [11]; the latter is reclassified as several *Porphyromonas* and *Prevotella* species [20]*.* Moreover, in other studies these bacteria are detected in IP samples [17, 18]. In addition to IP, *P. levii* is detected in DD lesions [22] and in an outbreak of necrotic vulvovaginitis [38]. Also, *T. pyogenes* is reported to occur in IP lesions [7, 11, 18]. In our study *T. pyogenes* was associated with a healing stage of IP and only a trend existed with acute IP, indicating that this pathogen has a secondary role in IP. Nevertheless, we were unable to establish an association between high morbidity and *P. levii* or *T. pyogenes*.

There are very few studies of the occurrence of treponemes in IP, but many concerning DD. Earlier studies revealed occurrence of *Spirochetes* in IP lesions [5, 7] but it remains uncertain whether the organisms were treponemes or not. Treponemes are regarded as the most important pathogens in DD [19, 22, 26, 39], and have been detected also in other hoof lesions, including toe necrosis, sole ulcer and white line disease [40, 41]. In our study, we detected *Treponema* group 2 and 3 in all disease categories, but more frequently in IP and in other hoof diseases; mainly DD. Interestingly all observed DD lesions were detected in herds of moderate morbidity (data not shown). To date ID and DD are not represented a major problem of cattle in Finland [42].

Of 217 cows sampled, 66 (30.4%) were currently being or had previously been treated with antimicrobials. It would have been unethical to leave the affected cows untreated until the sampling visit took place. Nevertheless, the possible effect of an antimicrobial treatment was taken into account in the analysis.

## Conclusion

In the current study, we investigated several bacteria in new type of outbreaks of IP and possible bacterial dissimilarities in herds with various morbidity. We could detect all studied bacteria in IP lesions either alone or in various combinations but observed bacteriological differences in herds with various morbidity. The most substantial finding was the presence of *F. necrophorum* in IP lesions, and *T. pyogenes* at the healing stage of IP. Our results also suggest that *D. nodosus* may play a role in the severity of the outbreak of IP. It is also quite apparent that a correct diagnosis of IP cannot be made based on a single bacteriologic sample without a clinical inspection.

Virulence factors of *F. necrophorum* isolates and transmission of hoof pathogens among and within farms may represent an important subject that merits further research.

## Abbreviations

DD: Digital dermatitis
ID: Interdigital dermatitis
IP: Interdigital phlegmon
